# Flapless Immediate Implant Placement With and Without Bone Grafting: A Systematic Review and Meta‐Analysis

**DOI:** 10.1002/cre2.70182

**Published:** 2025-07-23

**Authors:** Saeed Sadat Mansouri, Farhan Musaie, Abbas Mirzaei, Samad Samadizadeh, Salar Chaychi Salmasi, Tahereh Bitaraf

**Affiliations:** ^1^ Department of Periodontology, Faculty of Dentistry, Tehran Medical Sciences Islamic Azad University Tehran Iran; ^2^ Faculty of Dentistry Islamic Azad University Tehran Iran; ^3^ Library of Faculty of Dentistry Tehran University of Medical Sciences Tehran Iran; ^4^ Oral and Maxillofacial Surgeon Private Practice Tehran Iran; ^5^ Research Committee, REDDEN Center, School of Dentistry Tehran University of Medical Sciences Tehran Iran; ^6^ Dental Implant Research Center, Faculty of Dentistry, Tehran Medical Sciences Islamic Azad University Tehran Iran

**Keywords:** alveolar ridge augmentation, bone transplantation, flapless, socket graft

## Abstract

**Objectives:**

This study aims to evaluate the influence of bone grafting peri‐implant gap on the changes in hard and soft tissues following flapless immediate implantation.

**Material and Methods:**

A comprehensive research process was undertaken, including electronic searches of databases such as Embase, PubMed, Web of Science, and Cochrane, as well as a manual search. This approach identified relevant clinical studies until March 2024. Randomized controlled trials (RCTs) that compared flapless immediate implant placement with and without bone grafting were selected for qualitative assessment. Meta‐analyses were conducted where feasible. The protocol was registered in PROSPERO with code CRD42024499377, ensuring transparency and accountability in our research.

**Results:**

Out of 1065 articles, 5 RCTs were included, involving 179 patients who underwent placement of 179 immediate implants (92 with bone grafting and 87 without) with follow‐up periods ranging from 6 to 12 months. Analysis revealed no significant differences in horizontal bone changes within 0 to 3 mm apical to the bone crest between the two groups (weighted mean difference [WMD]: 0.147 (−0.012, 0.306), *I* ^2^: 0.0%; standardized mean difference [SMD]: 0.337 (−0.016, 0.690), *I* ^2^: 0.0%). However, significant differences were observed in horizontal bone changes at 4–5 mm apical to the reference point, favoring bone grafting (WMD: 0.484 (0.142, 0.826), *I* ^2^: 75.4%; SMD: 0.794 (0.402, 1.186), *I* ^2^: 36.6%). No significant differences were found in vertical midfacial soft tissue changes between the groups (WMD: 0.300 (−0.425, 1.024), *I* ^2^: 62.4%; SMD: 0.213 (−0.339, 0.764), *I* ^2^: 60.2%). Data limitations precluded meta‐analyses on additional outcomes such as mesial and distal papillary alterations and vertical bone level changes.

**Conclusion:**

Flapless immediate dental implants with a bone graft may enhance alveolar bone preservation, warranting further investigation.

## 1 Introduction

Maintaining the long‐term stability of hard tissues after tooth extraction and during immediate implant placement requires addressing challenges such as dimensional changes in bone and the buccal bone gap (Seyssens et al. 2020). Long‐term studies have shown alterations in the dimensions of the buccal bone after immediate implant placement, with some observations demonstrating limited or even complete loss of the buccal bone (Seyssens et al. 2020; Noelken et al. 2018). This can lead to soft tissue margin displacement and subsequent complications (Seyssens et al. 2020, 2022; Noelken et al. 2018). Additionally, esthetic issues associated with immediate implant placement, such as midfacial soft tissue recession and loss of contour, are frequently observed, particularly in cases where the buccal bone is compromised (Seyssens et al. 2020; Noelken et al. 2018). To enhance esthetics and preserve bone dimensions during immediate implant placement procedures, it is essential to employ strategies such as flapless techniques, bone grafting, and immediate provisionalization (Bakkali et al. 2021; Hassani et al. 2021; Pitman et al. 2022).

Flapless surgery effectively preserves bone integrity by preventing the exposure of underlying bone and minimizing disruption to the blood supply associated with mucoperiosteal flap elevation (Pitman et al. 2023; MeshkatAlsadat et al. 2022). A recent meta‐analysis reported that flapless techniques significantly reduce horizontal buccal bone alteration (0.48 mm) compared to flap‐based immediate implant placement, underscoring the benefits of flapless surgery for enhanced preservation of hard tissue (Pitman et al. 2023). Furthermore, flapless surgery offers advantages in wound stability and healing, especially when combined with regenerative approaches during immediate implant placement procedures, ultimately minimizing invasiveness and improving patient comfort (MeshkatAlsadat et al. 2022; Girlanda et al. 2019; Bittner et al. 2020).

Filling the buccal/labial gap during immediate dental implant placement can significantly enhance esthetic outcomes by promoting bone formation and preserving buccal bone thickness (Seyssens et al. 2022; Girlanda et al. 2019). A recent meta‐analysis confirmed a statistically significant reduction in horizontal buccal bone resorption with socket grafting. The mean reduction ranged from 0.37 to 1.07 mm with grafting, compared to 0.91–1.59 mm without, showing a 0.59 mm difference (Seyssens et al. 2022). Additionally, socket grafting effectively reduced apical recession of the midfacial soft tissue, with vertical soft tissue changes ranging from 0.58 to 0.94 mm following grafted immediate implant placement, compared to 0.92–1.69 mm without grafting, a 0.58 mm reduction (Seyssens et al. 2022). However, the efficacy of bone grafts in the buccal gap remains a subject of debate, as controlled clinical trials have yielded inconclusive results (Seyssens et al. 2022; Fettouh et al. 2023; Cardaropoli et al. 2014; Jacobs et al. 2020; Shahdad et al. 2020).

Recent systematic reviews have underscored the benefits of socket grafting (Seyssens et al. 2022) and flapless surgery (Pitman et al. 2023) in preserving horizontal buccal bone during immediate implant placement. However, a notable gap exists in the literature regarding the comparative effects of these grafting techniques on dimensional changes, particularly in the context of flapless immediate implant placement (Seyssens et al. 2022; Bakkali et al. 2021; Pitman et al. 2023). This systematic review and meta‐analysis were conducted to address this issue to compare outcomes of flapless immediate implant placement with and without bone grafting, focusing on both hard and soft tissue changes. The primary objective was to evaluate horizontal alveolar bone changes associated with flapless immediate implant placement with bone grafting versus without bone grafting. Secondary objectives included assessing vertical midfacial soft tissue changes and mesial and distal papillary tissue alterations.

## 2 Materials and Methods

This systematic review followed the PRISMA guidelines established by Page MJ et al (Page et al. 2021). The protocol for this review was registered in PROSPERO (CRD42024499377). Ethical approval was not necessary for this systematic review.

The PICOS criteria were defined as follows:

- Population: Patients undergoing flapless immediate implant placement.
- Intervention: Bone grafting.
- Comparison: No bone grafting.
- Outcomes:
*Primary outcomes*: Horizontal bone changes were assessed by CBCT, measuring the
  distance between the buccal and lingual alveolar bone surfaces and/or the amount of bone
  formed labial/buccal to the implant at different levels (0–5 mm) below the bone crest, both at baseline and follow‐up.
*Secondary outcomes*: Vertical midfacial soft tissue changes were evaluated by measuring from the free gingival margin using a prefabricated device, such as a stent, at baseline, the final follow‐up, and mesial and distal papillary changes.
- Study design: Randomized controlled trials.

### 2.1 Eligibility Criteria

To be considered for inclusion in this review, studies must meet the following requirements. They should be randomized controlled trials that compare flapless immediate implant placement with and without bone grafting. All selected studies must assess the primary outcome; however, evaluating secondary outcomes is not mandatory.

Studies will be excluded if they follow a non‐randomized controlled trial design or focus on flapless immediate implant placement with bone grafting without a comparative control group.

### 2.2 Search Strategy and Information Sources

Two reviewers, AM and FM, independently conducted a comprehensive literature search through electronic databases and manual searches to identify relevant clinical investigations. The search was performed in Embase, PubMed, Web of Science, and Cochrane databases up to March 2024. The detailed search strategy for PubMed is outlined below, while the methods for the other databases are provided in the appendix (Supplementary Table 1). The search terms used in PubMed included flapless AND (“bone transplantation”[mh] OR “bone regeneration”[mh] OR “alveolar ridge augmentation”[mh] OR graft*[tiab] OR “bone transplantation*”[tiab] OR “bone regeneration*”[tiab] OR osteoconduction[tiab] OR “alveolar ridge augmentation*”[tiab] OR “alveolar ridge preservation”[tiab] OR “socket preservation”[tiab] OR “socket graft*”[tiab] OR “bone augmentation”[tiab] OR “bone replacement graft”[tiab] OR “bovine bone”[tiab] OR xenograft[tiab] OR “mandibular ridge augmentation*”[tiab] OR “maxillary ridge augmentation*”[tiab] OR “bone patellar tendon bone grafting”[tiab] OR “bone reimplantation*”[tiab] OR “bone‐patellar tendon‐bone grafting”[tiab] OR “ridge augmentation procedure”[tiab]).

Two reviewers (S.C.S. and F.M.) independently assessed the eligibility of all studies according to predefined inclusion and exclusion criteria. This evaluation was initially conducted at the title level, followed by an assessment at the abstract level. Articles that met the requirements at the abstract level were then retrieved for full‐text review. In cases of uncertainty at either the title or abstract level, further examination was performed to ensure no relevant studies were overlooked. Any disagreements during the full‐text review process were resolved through discussion with a third reviewer (S.S.M.).

We conducted forward and backward citation searches on the included studies. In addition, we manually searched Clinical Implant Dentistry and Related Research, Clinical Oral Implants Research, and International Journal of Periodontics and Restorative Dentistry to ensure all relevant studies were identified. We utilized Scopus (scopus.com) for these two additional searches.

### 2.3 Risk of Bias Assessment

The reviewers (A.M., F.M.) independently assessed the quality of the included randomized controlled trials using the Revised Cochrane Risk‐of‐bias Tool (RoB 2) (Sterne et al. 2019). The review assessed bias in five key domains: (1) bias resulting from randomization procedures, (2) bias stemming from deviations in intended interventions, (3) bias due to incomplete outcome data, (4) bias in the measurement of outcomes, and (5) bias in the selection of reported results. Each domain was evaluated for low, unclear, or high risk of bias. Any discrepancies identified during the quality assessment were resolved through discussion with the third reviewer (T.B.). Additionally, the risk of bias between studies was evaluated using the Egger test and funnel plot to detect publication bias (Rokn et al. 2018).

### 2.4 Data Extraction and Statistical Analysis

Two independent assessors (S.S. and F.M.) utilized a specific data extraction form (Table 1). Using a distribution‐based approach, the present systematic review and meta‐analysis established the minimum clinically significant difference (MCID) for bone changes at 0.3 mm, equivalent to half a standard deviation (Kermanshah et al. 2023).

**Table 1:** Features of the randomized controlled trials included in the study.

Author/follow‐up (Month)	Number of patients/implant; implant site distribution		Eligibility criteria	Mean age (years)	Buccal bone gaps	Grafting material	Implant placement (freehand/guided), implant type (bone/tissue level)	Type of provisionalization	Definitive restoration	Horizontal bone measurements methods	Included Study Outcomes
	FIIBG	FII									
Fettouh et al. (2023)/12 months	20/20 Ce: 5 La: 5 Ca: 1 P1: 8 P2: 1	20/20 Ce: 3 La: 2 Ca: ‐ P1: 11 P2: 4	Nonrestorable maxillary teeth; thick gingiva; intact thin labial bone ≤ 1 mm width, 7 mm length; palatal bone ≥ 6 mm length; apical stability ≥ 35 Ncm; mid‐crest socket ≥ 5 mm, nonsmoker	36.45	labial gap at least 1.5 mm	DBBM (Bio‐Oss, Geistlich Pharma AG, Switzerland)	Zimmer TSV; Freehand; Bone Level	Custom healing abutment (Filtek Flowable) on cylindrical abutment at implantation.	Final crown placed after 1 year.	CBCT radiographs pre‐extraction and follow‐up	HBC
Jacobs et al. (2020)/10 months	19/19 Ce: 8 La: 4 Ca: 4 P1: 3	14/14 Ce:7 La: 5 Ca:2 P1: 0	Single maxillary tooth (P1–P1), intact labial bone, no smoking for 1 year	48	NR	DBBM (Bio‐Oss) + Collagen Plug (Zimmer Dental)	OsseoSpeed TX (Dentsply Sirona); Guided; Bone Level;	Bonded pontics with resin nanoceramic and healing abutment, immediately postimplantation. Screw‐retained provisional restoration with abutment‐level temporary cylinders, 12 weeks later.	Lithium disilicate crown on gold‐shaded titanium CAD/CAM abutment, 7 months postimplantation	CBCT radiographs pre‐extraction and follow‐up	HBC; VMSC; MPC; DPC
Bittner et al. (2020)/12 months	16/16 Ce & La: 7 Ca: 0 P1: 9	16/16 Ce & La: 10 Ca: 1 P1: 5	Single maxillary tooth (P1–P1), intact labial bone, no smoking in the last 6 months	52.3	Test: 2.9 ± 1.3 Control 3.1 ± 0.9	C‐DBBM (Bio‐Oss Collagen)	Zimmer Biomet Certain; Freehand; Bone Level	Full‐contour screw‐retained provisional or PMMA healing abutment after implant placement	Screw‐retained porcelain‐fused‐to‐metal restoration, 6 months postimplantation	Digital superimposed casts from baseline to 12 months	HBC; VMSC; MPC; DPC
Girlanda et al. (2019)/6 months	11/11 incisor	11/11 incisor	Single maxillary incisor; intact labial bone; nonsmoker or ex‐smoker	40	test 2.55 ± 0.52 Control 2.45 ± 0.52	C‐DBBM (Bio‐Oss Collagen)	Biomet 3i Full Osseotite; Freehand; Bone	Temporary abutment for 3 months	Fixed prosthesis on stock abutment at 3 months	CBCT preoperative and at 6 months	HBC; VMSC; MPC; DPC
Cardaropoli et al. (2014)/12 months	26/26 Ce: l4 La: 7 Ca: 0 P1: 7 P2: 8	26/26 Ce: 2 La: 2 Ca: 2 P1:12 P2: 8	Single maxillary/mandibular anterior tooth; ≤ 10 cigarettes/day; no smoking pre‐ and post‐surgery	43	NR	C‐DBBM (Bio‐Oss Collagen) + Collagen membrane (BioGide, Geistlich)	Biomet 3i Osseotite; Freehand; Bone Level	Provisional crown replacing healing abutment after 3 months	Porcelain crown placed after 5 months	Cast‐based measurement with silicone stent	HBC

Abbreviations: Ant, anterior; Ca, Canine; CBCT, cone beam computed tomography; Ce, central incisor; C‐DBBM, deproteinized bovine bone mineral with 10% collagen; DBBM, deproteinized bovine bone mineral; DPC, distal papillary changes; FIIBG, flapless immediate implant with bone grafting; FII, flapless immediate implant; HBC, horizontal bone changes; La, lateral incisor; MPC, mesial papillary changes; NR, not reported; P1, first premolar; P2, second premolar; PMMA, polymethyl methacrylate; Post, Posterior; Q, implant diameter; VMSC, vertical midfacial soft tissue changes.

Weighted mean difference (WMD) and standardized mean difference (SMD) were utilized to evaluate quantitative primary and secondary outcomes. The differences in the horizontal alveolar bone changes at the last follow‐up were analyzed for meta‐analysis to avoid excluding certain randomized controlled trials that did not report baseline and follow‐up MBL levels for all study groups. The size of the effect (SMD) was interpreted as small (0.2–0.49), medium (0.50–0.79), large (0.80–0.99), and very large effect ( ≥ 1.0). The conclusive result was reached based on the narrow confidence intervals aligning with one of the specified ranges, a large effect size, and a clinically significant difference (Kraemer et al. 2003).

The meta‐analysis was performed using Stata version 14.2 with a random‐effects model. Heterogeneity was evaluated through the Cochrane Q test, *p*‐values, and *I* ^2^ statistic. Subgroup analysis was undertaken to investigate potential sources of heterogeneity, particularly emphasizing the quality of randomized controlled trials. Additionally, a sensitivity analysis was conducted utilizing the metaninf module with the one‐out removed method to assess the robustness of the findings (Rokn et al. 2018).

## 3 Results

### 3.1 Search and Description of Included Studies

**Figure 1:**
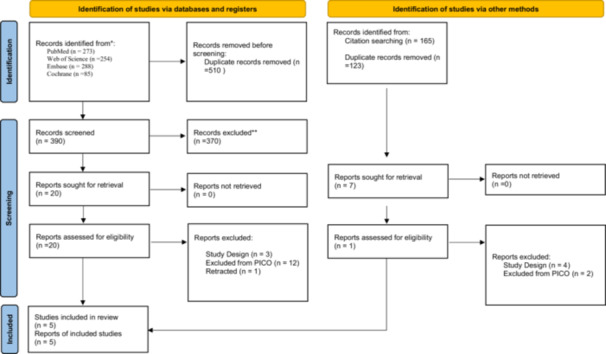
PRISMA flowchart. Electronic search results.

Initially, 1065 studies were found in the database. After removing duplicates and reviewing titles and abstracts, 20 articles were selected for further examination. After evaluating the full texts, five articles were included for statistical analysis (Girlanda et al. 2019; Bittner et al. 2020; Fettouh et al. 2023; Cardaropoli et al. 2014; Jacobs et al. 2020) (Figure 1), and others were excluded (Supplementary Table 2) (MeshkatAlsadat et al. 2022; Bungthong et al. 2022; Amato et al. 2018; Elaskary et al. 2022; Fernandes et al. 2021; Naji et al. 2021; Kumar et al. 2021; Ferrantino et al. 2021; Abd‐Elrahman et al. 2020; Grassi et al. 2019; Natto et al. 2017; Shahdad et al. 2020; Tarnow et al. 2014; Cardaropoli et al. 2018; Paknejad et al. 2017; Bottini et al. 2012). The characteristics of the five included RCTs are detailed in Table 1. By March 2024, 179 implants had been received by 179 patients, with a mean age of 44.93 years. These implants consisted of 92 flapless immediate implants with bone graft (FIIBG) and 87 flapless immediate implants (FII).

**Figure 2:**
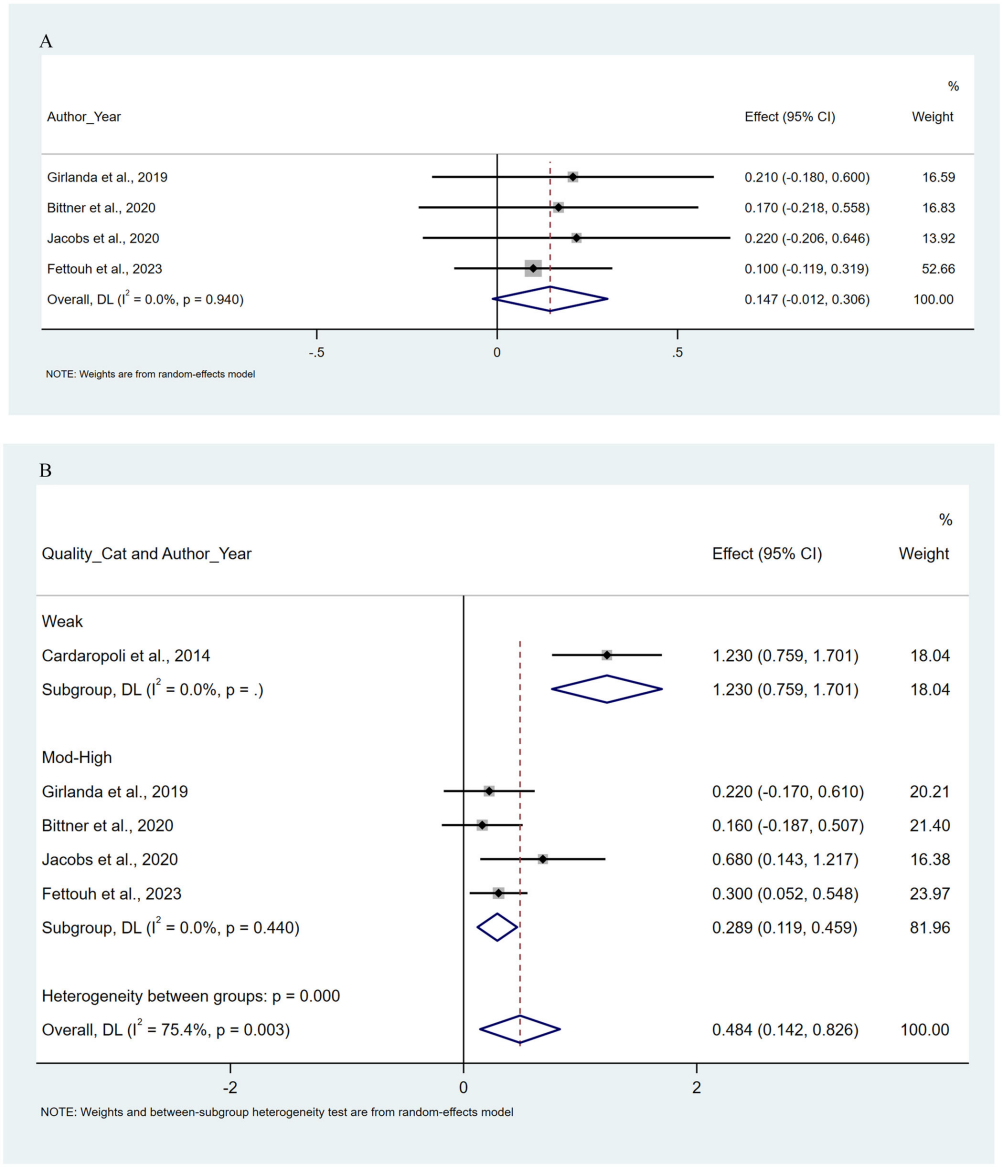
Forest plots. Difference of horizontal bone changes of FIIBG versus FII, weighted mean difference (WMD) in (A) 0–3 mm, (B) 4–5 mm by quality of study subgroup analysis.

Case selection in all included studies was limited to intact buccal/facial bone. The four articles focused on single implants placed in the anterior maxilla or both anterior jaws, evaluated in the Cardaropoli study. Deproteinized bovine bone mineral (DBBM) was utilized for grafting the peri‐implant gap in two studies (Fettouh et al. 2023; Jacobs et al. 2020), while deproteinized bovine bone mineral with 10% collagen (C‐DBBM) was used in three studies (Girlanda et al. 2019; Bittner et al. 2020; Cardaropoli et al. 2014). In the four articles, a membrane was not utilized with FIIBG; however, Cardaropoli and colleagues (2014) employed a membrane to cover the buccal gap when inserting graft material. Three studies implemented immediate restoration (Girlanda et al. 2019; Bittner et al. 2020; Fettouh et al. 2023), and none of the five studies utilized a connective tissue graft. Subgroup analysis based on methodological quality revealed significant differences between moderate‐high and low‐quality groups (*p* value for interaction: 0.006), as depicted in Figure 2.

### 3.2 Primary Outcome Variable: Alterations in the Horizontal Alveolar Bone Changes

Five randomized controlled trials examined horizontal bone alterations in flapless immediate implant placement with or without bone grafting at baseline and at the 6‐ to 12‐month follow‐up (Girlanda et al. 2019; Bittner et al. 2020; Fettouh et al. 2023; Cardaropoli et al. 2014; Jacobs et al. 2020).

Three included studies used cone‐beam computed tomography (CBCT) images to assess changes in bone dimensions (Girlanda et al. 2019; Fettouh et al. 2023; Jacobs et al. 2020). In contrast, Cardaropoli et al. (2014) employed dental casts and silicone stents, while Bittner et al. (2020) utilized digital superimposed casts. Four studies assessed horizontal buccal bone changes at 0–3 mm apical to the reference point, whereas all five studies evaluated these changes at 4–5 mm apical to the reference. Cardaropoli and colleagues (2014) did not include an evaluation point within the 0–3 mm range. The meta‐analysis included data from five randomized controlled trials involving 179 immediately placed flapless implants (92 with simultaneous bone grafting and 87 without). There were no statistically significant differences in horizontal bone changes at the 0–3 mm apical to the level of the reference point between FIIBG and FII (WMD: 0.147 (−0.012, 0.306), *I* ^2^: 0.0%; SMD: 0.337 (CI: −0.016, 0.690), *I* ^2^: 0.0%). There were statistically significant differences in horizontal bone changes at the 4–5 mm apical to the level of the reference point, preferring Bone grafting (WMD: 0.484 (0.142, 0.826), *I* ^2^: 75.4%; SMD: 0.794 (CI: 0.402, 1.186), *I* ^2^: 36.6%) (Figure 2).

### 3.3 Secondary Outcome Variables

#### 3.3.1 Vertical Midfacial Soft Tissue Changes

**Figure 3:**
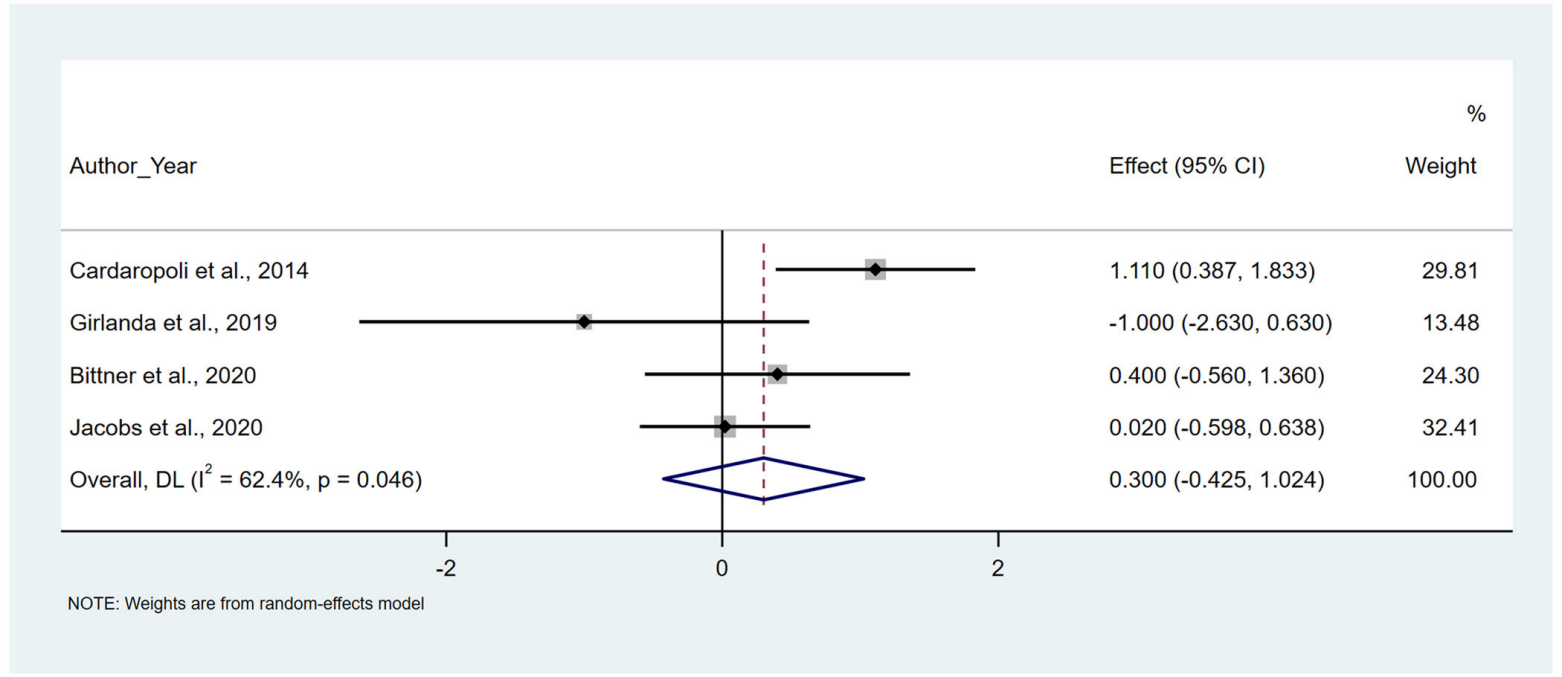
Forest plots. Difference of vertical midfacial soft tissue changes of FIIBG versus FII, weighted mean difference (WMD).

Figure 3 presents the secondary outcome variables reported in the included studies, focusing on vertical midfacial soft tissue changes around flapless immediate implants. Studies investigated the vertical soft tissue level at the buccal aspect of immediate implants using various methods. Some studies utilized individualized stents and periodontal probing to assess soft tissue levels (Girlanda et al. 2019; Bittner et al. 2020). Cardaropoli et al. (2014) used cast models for evaluation, and Jacobs et al. (2020) measured it with a periodontal probe and reference fixed points on adjacent teeth.

The meta‐analysis indicated no significant difference in apical migration of the midfacial soft tissue level in four included studies (Girlanda et al. 2019; Bittner et al. 2020; Cardaropoli et al. 2014; Jacobs et al. 2020) measured the Vertical midfacial soft tissue changes at the time of tooth extraction and during a follow‐up surgery between 6 and 12 months after the implant was placed. There were no statistically significant differences in vertical midfacial soft tissue changes between FIIBG and FII (WMD: 0.300 (−0.425, 1.024), *I* ^2^: 62.4%; SMD: 0.213 (CI: −0.339, 0.764), *I* ^2^: 60.2%) (Figure 3).

#### 3.3.2 Mesial and Distal Papillary Changes

The three studies examined vertical soft tissue changes following flapless immediate implant with bone graft and flapless immediate implant, showing varying results in mean changes at mesiobuccal and distobuccal sites. While the meta‐analysis was not conducted due to limited studies, significant differences favoring the test group were found in the distal papilla in two included studies (Girlanda et al. 2019; Bittner et al. 2020) but not in the mesial papilla in Jacobs et al.'s (2020) study. Inconsistencies were observed when comparing soft tissue changes at mesial papillae among the three studies, with Girlanda et al.'s (2019) study showing a significantly higher soft tissue height in the test group at the mesiobuccally.

#### 3.3.3 Risk of Bias Assessment and Sensitivity Analysis

Table 2 illustrates the evaluation of study quality using the RoB 2 framework. The studies conducted by Girlanda et al. (2019) and Fettouh et al. (2023) were deemed to be of the highest quality, as all five domains were rated as low risk of bias. Conversely, studies by Jacobs et al. (2020) had at least one domain classified as high risk of bias. The remaining studies also raised concerns regarding bias (Bittner et al. 2020; Cardaropoli et al. 2014).

**Table 2:** Evaluation of quality of randomized controlled trials using the revised Cochrane risk of bias tool.

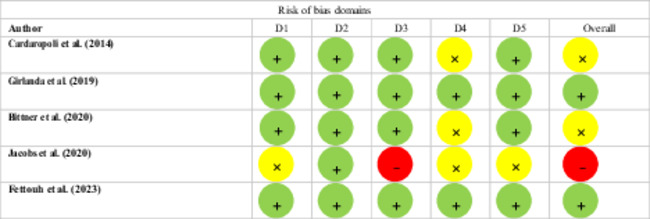

*Note:* Assessment of bias was categorized as red for high risk, yellow for some concerns, and green for low risk. The domains evaluated include D1 for bias related to randomization, D2 for bias arising from deviations in the intervention, D3 for bias due to missing outcome data,

D4 for bias in outcome measurement, and D5 for bias in result selection.

**Figure 4:**
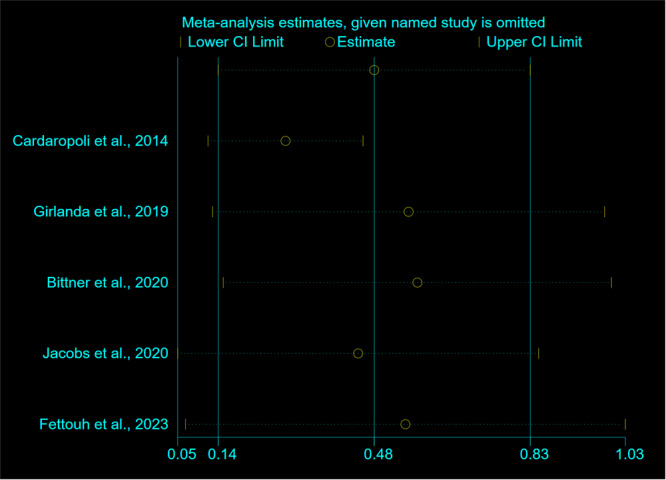
One‐out remove graph in a weighted mean difference of horizontal bone changes of FIIBG versus FII (developed by metaninf module of Stata).

The funnel plot of WMD of MBLs showed a diverse range of points, but the publication bias was considered insignificant according to Egger's test result (*Z*: −0.98, *p* = 0.199). The analysis using the one‐out‐removed method revealed that excluding one study did not significantly alter the overall results compared to including all studies. Therefore, the combined results from the RCTs were not heavily influenced by any single study (Figure 4).

## 4 Discussion

This study revealed that Flapless Immediate Implant with Bone Grafting and Flapless Immediate Implant demonstrated comparable outcomes in horizontal bone and midfacial soft tissue changes. However, FIIBG exhibited a statistically significant decrease in horizontal bone changes at the reference point's 4–5 mm apical level compared to FII. Further study is needed to fully evaluate the impact of bone grafting on alveolar bone preservation.

Recent studies, aligned with Delphi‐based methodologies (Schrover et al. 2017), underscore the critical objective of achieving zero bone loss (Linkevicius 2019), as evidenced by prior research. The included articles report labial/buccal bone thickness of less than 1 mm, highlighting the necessity of addressing even minimal bone resorption (Heimes et al. 2021). At the 4‐ to 5‐mm level, FIIBG has demonstrated a clinically significant difference (Schrover et al. 2017), achieving a 0.48 mm horizontal bone change and a medium‐size difference in the standardized mean difference (SMD), favoring this approach within the Delphi framework (Schrover et al. 2017) and supported by analysis of the high‐moderate quality subgroup. The subgroup analysis effectively reduced the severe heterogeneity to 0% (Kraemer et al. 2003), while the 0.28 mm difference in horizontal bone change remains within the minimal clinically significant difference (MCID) threshold (Schrover et al. 2017), reaffirming the clinical relevance of even slight bone loss. Notably, severe heterogeneity correlated with a lower‐quality subgroup. These findings emphasize the fundamental role of bone grafting at all peri‐implant levels in maintaining adequate bone thickness and ensuring long‐term stability. However, further research is warranted to substantiate these findings.

Bone grafting can be essential in preserving the buccal bone wall, reducing horizontal resorption, and providing a scaffold for new bone growth, which enhances healing and implant integration. Reduced resorption in the 0–3 mm apical to the reference point is due to the stability and regenerative capacity of the crestal bone (Hu et al. 2021; Sai Priyanka et al. 2024). However, in the 4‐ to 5‐mm apical to the reference point, anatomical discrepancies, such as the labial inclination of natural roots versus the palatal positioning of implants, create buccal gaps that require grafting. This middle‐third region is biomechanically vulnerable and more prone to functional stress and resorption (Hu et al. 2021; Sai Priyanka et al. 2024), highlighting the need for further research on grafting in flapless immediate implant procedures.

CBCT offers superior spatial resolution, reduced radiation exposure, and cost‐effectiveness, establishing it as a reliable imaging modality for evaluating changes in bone dimensions (Fettouh et al. 2023). Additionally, its three‐dimensional imaging capability significantly enhances the diagnostic accuracy of peri‐implant tissues in the buccolingual dimension, addressing the inherent limitations of traditional two‐dimensional radiographic techniques, and enables the long‐term, indirect evaluation of horizontal bone changes after second‐stage surgery (Hu et al. 2021; Sai Priyanka et al. 2024). Previous studies employing direct evaluation methods, including surgical re‐entry during short follow‐up periods, generally corroborate CBCT findings, affirming its precision (Chen et al. 2007; Sanz et al. 2017). However, measurement variations observed in casts may be attributed to post‐setting shrinkage, which contributes to the heterogeneity in the present findings (Cardaropoli et al. 2014; Hong et al. 2019; Nemati Anaraki et al. 2019). This potential source of heterogeneity was addressed through subgroup analyses, reinforcing CBCT's reliability in clinical assessments, particularly in evaluating buccolingual bone width over extended follow‐up periods.

The findings demonstrated negligible publication bias, with no individual study significantly impacting the overall results. However, variability in follow‐up durations, ranging from 6 to 12 months, remains a notable limitation of the present analysis. Sensitivity analyses confirmed that shorter follow‐up durations did not alter the outcomes, alleviating concerns regarding this variability. To draw more definitive conclusions, there is an evident need for high‐quality, long‐term randomized clinical trials with standardized follow‐up durations and comprehensive outcome reporting, ensuring the robustness and reliability of future findings.

## 5 Conclusion

Our findings suggest that flapless immediate dental implants with a bone graft may improve alveolar bone preservation. Further studies are needed to confirm these results.

## Author Contributions

Saeed Sadat Mansouri, Farhan Musaie, Abbas Mirzaei, Samad Samadizadeh, Salar Chaychi Salmasi, and Tahereh Bitaraf collaborated on the design, execution, and interpretation of the study. They also drafted and revised the manuscript to ensure its precision. Farhan Musaie and Abbas Mirzaei focused on data extraction and methodology, while Tahereh Bitaraf played a crucial role in analyzing and interpreting the data. All authors approved the final version of the manuscript before submission.

## Conflicts of Interest

The authors declare no conflicts of interest.

## Supporting information


**Supplementary Table 1:**Search Strategy (Search Date: March 30, 2024).
**Supplementary Tables 2.** Description of excluded studies.

## Data Availability

The data that support the findings of this study are available from the corresponding author upon reasonable request.

## References

[cre270182-bib-0001] Abd‐Elrahman, A. , M. Shaheen , N. Askar , and M. Atef . 2020. “Socket Shield Technique vs Conventional Immediate Implant Placement With Immediate Temporization. Randomized Clinical Trial.” Clinical Implant Dentistry and Related Research 22, no. 5: 602–611. 10.1111/cid.12938. 32757311

[cre270182-bib-0002] Amato, F. , G. Polara , and G. A. Spedicato . 2018. “Tissue Dimensional Changes in Single‐Tooth Immediate Extraction Implant Placement in the Esthetic Zone: A Retrospective Clinical Study.” International Journal of Oral & Maxillofacial Implants 33, no. 2: 439–447. 10.11607/jomi.6146. 29534133

[cre270182-bib-0003] Bakkali, S. , M. Rizo‐Gorrita , M. M. Romero‐Ruiz , J. L. Gutiérrez‐Pérez , D. Torres‐Lagares , and M. Á. Serrera‐Figallo . 2021. “Efficacy of Different Surgical Techniques for Peri‐Implant Tissue Preservation in Immediate Implant Placement: A Systematic Review and Meta‐Analysis.” Clinical Oral Investigations 25, no. 4: 1655–1675. 10.1007/s00784-021-03794-y. 33515121

[cre270182-bib-0004] Bittner, N. , L. Planzos , A. Volchonok , D. Tarnow , and U. Schulze‐Späte . 2020. “Evaluation of Horizontal and Vertical Buccal Ridge Dimensional Changes After Immediate Implant Placement and Immediate Temporization With and Without Bone Augmentation Procedures: Short‐Term, 1‐Year Results. A Randomized Controlled Clinical Trial.” International Journal of Periodontics and Restorative Dentistry 40, no. 1: 83–93. 10.11607/prd.4152. 31815977

[cre270182-bib-0005] Bottini, L. P. , L. Ricci , A. Piattelli , V. Perrotti , and G. Iezzi . 2012. “Bucco‐Lingual Crestal Bone Changes Around Implants Immediately Placed in Fresh Extraction Sockets in Association or Not With Porcine Bone: A Non‐Blinded Randomized Controlled Trial in Humans [Retracted in: *Journal of Periodontology*. 2017 Dec;88(12):1374. 10.1902/jop.2017.1712002].” Journal of Periodontology, ahead of print, October 29. 29166847

[cre270182-bib-0006] Bungthong, W. , P. Amornsettachai , P. Luangchana , B. Chuenjitkuntaworn , and S. Suphangul . 2022. “Bone Dimensional Change Following Immediate Implant Placement in Posterior Teeth With CBCT: A 6‐Month Prospective Clinical Study.” Molecules 27, no. 3: 608. 10.3390/molecules27030608. 35163869 PMC8838578

[cre270182-bib-0007] Cardaropoli, D. , L. Gaveglio , E. Gherlone , and G. Cardaropoli . 2014. “Soft Tissue Contour Changes at Immediate Implants: A Randomized Controlled Clinical Study.” International Journal of Periodontics and Restorative Dentistry 34, no. 5: 631–637. 10.11607/prd.1845. 25171033

[cre270182-bib-0008] Cardaropoli, D. , L. Tamagnone , A. Roffredo , A. De Maria , and L. Gaveglio . 2018. “Alveolar Ridge Preservation Using Tridimensional Collagen Matrix and Deproteinized Bovine Bone Mineral in the Esthetic Area: A CBCT and Histologic Human Pilot Study.” International Journal of Periodontics & Restorative Dentistry 38, no. Suppl: s29–s35. 10.11607/prd.3702. 30118530

[cre270182-bib-0009] Chen, S. T. , I. B. Darby , and E. C. Reynolds . 2007. “A Prospective Clinical Study of Non‐Submerged Immediate Implants: Clinical Outcomes and Esthetic Results.” Clinical Oral Implants Research 18, no. 5: 552–562. 10.1111/j.1600-0501.2007.01388.x. 17608739

[cre270182-bib-0010] Elaskary, A. , H. Abdelrahman , H. H. Elsabagh , and G. I. El‐Kimary . 2022. “Does Grafting the Jumping Gap in Immediately Placed Anterior Implants Using Vestibular Socket Therapy Influence the Labial Bone Thickness?” Journal of Oral and Maxillofacial Surgery 80, no. 8: 1398–1407. 10.1016/j.joms.2022.05.001. 35688272

[cre270182-bib-0011] Fernandes, D. , S. Nunes , G. López‐Castro , T. Marques , J. Montero , and T. Borges . 2021. “Effect of Customized Healing Abutments on the Peri‐Implant Linear and Volumetric Tissue Changes at Maxillary Immediate Implant Sites: A 1‐Year Prospective Randomized Clinical Trial.” Clinical Implant Dentistry and Related Research 23, no. 5: 745–757. 10.1111/cid.13044. 34423560

[cre270182-bib-0012] Ferrantino, L. , A. Camurati , P. Gambino , et al. 2021. “Aesthetic Outcomes of Non‐Functional Immediately Restored Single Post‐Extraction Implants With and Without Connective Tissue Graft: A Multicentre Randomized Controlled Trial.” Clinical Oral Implants Research 32, no. 6: 684–694. 10.1111/clr.13733. 33638216

[cre270182-bib-0013] Fettouh, A. I. A. , N. A. Ghallab , K. A. Ghaffar , et al. 2023. “Bone Dimensional Changes After Flapless Immediate Implant Placement With and Without Bone Grafting: Randomized Clinical Trial.” Clinical Implant Dentistry and Related Research 25, no. 2: 271–283. 10.1111/cid.13178. 36596471

[cre270182-bib-0014] Girlanda, F. F. , H. S. Feng , M. G. Corrêa , et al. 2019. “Deproteinized Bovine Bone Derived With Collagen Improves Soft and Bone Tissue Outcomes in Flapless Immediate Implant Approach and Immediate Provisionalization: A Randomized Clinical Trial.” Clinical Oral Investigations 23, no. 10: 3885–3893. 10.1007/s00784-019-02819-x. 30693399

[cre270182-bib-0015] Grassi, F. R. , R. Grassi , B. Rapone , G. Alemanno , A. Balena , and Z. Kalemaj . 2019. “Dimensional Changes of Buccal Bone Plate in Immediate Implants Inserted Through Open Flap, Open Flap and Bone Grafting and Flapless Techniques: A Cone‐Beam Computed Tomography Randomized Controlled Clinical Trial.” Clinical Oral Implants Research 30, no. 12: 1155–1164. 10.1111/clr.13528. 31461183

[cre270182-bib-0016] Hassani, A. , M. Hassani , and T. Bitaraf . 2021. “Immediate Vs Delayed Restorations of Immediately Placed Single Implants in the Anterior Maxilla: A Nonrandomized Clinical Study.” International Journal of Oral & Maxillofacial Implants 36, no. 6: 1159–1164. 10.11607/jomi.8947. 34919616

[cre270182-bib-0017] Heimes, D. , E. Schiegnitz , R. Kuchen , P. W. Kämmerer , and B. Al‐Nawas . 2021. “Buccal Bone Thickness in Anterior and Posterior Teeth‐A Systematic Review.” Healthcare 9, no. 12: 1663. 10.3390/healthcare9121663. 34946389 PMC8700878

[cre270182-bib-0018] Hong, H. R. , C. Y. Chen , D. M. Kim , and E. E. Machtei . 2019. “Ridge Preservation Procedures Revisited: A Randomized Controlled Trial to Evaluate Dimensional Changes With Two Different Surgical Protocols.” Journal of Periodontology 90, no. 4: 331–338. 10.1002/JPER.18-0041. 30367733

[cre270182-bib-0019] Hu, K. F. , S. W. Lin , Y. C. Lin , et al. 2021. “Using Cone‐Beam Computed Tomography to Assess Changes in Alveolar Bone Width Around Dental Implants at Native and Reconstructed Bone Sites: A Retrospective Cohort Study.” Journal of Personalized Medicine 11, no. 10: 1011. 10.3390/jpm11101011. 34683152 PMC8537892

[cre270182-bib-0020] Jacobs, B. , H. Zadeh , I. De Kok , and L. Cooper . 2020. “A Randomized Controlled Trial Evaluating Grafting the Facial Gap at Immediately Placed Implants.” International Journal of Periodontics and Restorative Dentistry 40, no. 3: 383–392. 10.11607/prd.3774. 32130284

[cre270182-bib-0021] Kermanshah, H. , A. Keshtkar , A. Hassani , and T. Bitaraf . 2023. “Comparing Short Implants to Standard Dental Implants: A Systematic Review and Meta‐Analysis of Randomized Controlled Trials With Extended Follow‐Up.” Evidence‐Based Dentistry 24, no. 4: 192–193. 10.1038/s41432-023-00924-1. 37568011

[cre270182-bib-0022] Kraemer, H. C. , G. A. Morgan , N. L. Leech , J. A. Gliner , J. J. Vaske , and R. J. Harmon . 2003. “Measures of Clinical Significance.” Journal of the American Academy of Child & Adolescent Psychiatry 42, no. 12: 1524–1529. 10.1097/00004583-200312000-00022. 14627890

[cre270182-bib-0023] Kumar, P. R. , J. Vikram , U. Kher , A. Tunkiwala , and H. Sawhney . 2021. “Pink Esthetic and Radiological Scores Around Immediate Implants Placed in the Esthetic Zone – Socket‐Shield Technique Versus Immediate Conventional Technique: A Pilot Study.” Journal of Indian Society of Periodontology 25, no. 6: 510–517. 10.4103/jisp.jisp_278_20. 34898917 PMC8603788

[cre270182-bib-0024] Linkevicius, T. 2019. “Is Zero Bone Loss a Posssibility When Placing Implants?” International Dentistry African Edition 8, no. 4: 34–36.

[cre270182-bib-0025] MeshkatAlsadat, M. , A. Hassani , T. Bitaraf , and S. C. Salmasi . 2022. “Dimensional Changes of Peri‐Implant Tissue Following Immediate Flapless Implant Placement and Provisionalization With or Without Xenograft in the Anterior Maxilla: A Study Protocol for a Randomized Controlled Trial.” Trials 23, no. 1: 960. 10.1186/s13063-022-06918-1. 36435819 PMC9701367

[cre270182-bib-0026] Naji, B. M. , S. S. Abdelsameaa , A. Y. Alqutaibi , and W. M. Said Ahmed . 2021. “Immediate Dental Implant Placement With a Horizontal Gap More Than Two Millimetres: A Randomized Clinical Trial.” International Journal of Oral and Maxillofacial Surgery 50, no. 5: 683–690. 10.1016/j.ijom.2020.08.015. 32951965

[cre270182-bib-0027] Natto, Z. S. , A. Parashis , B. Steffensen , R. Ganguly , M. D. Finkelman , and Y. N. Jeong . 2017. “Efficacy of Collagen Matrix Seal and Collagen Sponge on Ridge Preservation in Combination With Bone Allograft: A Randomized Controlled Clinical Trial.” Journal of Clinical Periodontology 44, no. 6: 649–659. 10.1111/jcpe.12722. 28303642

[cre270182-bib-0028] Nemati Anaraki, S. , H. Kazemi , Z. GHafari , Z. Naser , and T. Bitaraf . 2019. “In‐Vitro Comparative Study of the Effect of Four Finishing and Polishing Tools on Surface Roughness of a Microhybrid Resin Composite.” Journal of Research in Dental and Maxillofacial Sciences 4, no. 2: 26–31. 10.29252/jrdms.4.2.26.

[cre270182-bib-0029] Noelken, R. , M. Moergel , M. Kunkel , and W. Wagner . 2018. “Immediate and Flapless Implant Insertion and Provisionalization Using Autogenous Bone Grafts in the Esthetic Zone: 5‐Year Results.” Clinical Oral Implants Research 29, no. 3: 320–327. 10.1111/clr.13119. 29537706

[cre270182-bib-0030] Page, M. J. , J. E. McKenzie , P. M. Bossuyt , et al. 2021. “The PRISMA 2020 Statement: An Updated Guideline for Reporting Systematic Reviews.” BMJ 372, no. 71: n71. 10.1136/bmj.n71. 33782057 PMC8005924

[cre270182-bib-0031] Paknejad, M. , S. Akbari , H. Aslroosta , M. Panjnoush , and S. Hajheidary . 2017. “Effect of Flapless Immediate Implantation and Filling the Buccal Gap With Xenograft Material on the Buccal Bone Level: A Randomized Clinical Trial.” Journal of Dentistry (Tehran) 14, no. 6: 344–351. PMC6015591 29942329

[cre270182-bib-0032] Pitman, J. , V. Christiaens , J. Callens , et al. 2023. “Immediate Implant Placement With Flap or Flapless Surgery: A Systematic Review and Meta‐Analysis.” Journal of Clinical Periodontology 50, no. 6: 755–764. 10.1111/jcpe.13795. 36843361

[cre270182-bib-0033] Pitman, J. , L. Seyssens , V. Christiaens , and J. Cosyn . 2022. “Immediate Implant Placement With or Without Immediate Provisionalization: A Systematic Review and Meta‐Analysis.” Journal of Clinical Periodontology 49, no. 10: 1012–1023. 10.1111/jcpe.13686. 35734911

[cre270182-bib-0034] Rokn, A. R. , A. Keshtkar , A. Monzavi , K. Hashemi , and T. Bitaraf . 2018. “Comparing Short Dental Implants to Standard Dental Implants: Protocol for a Systematic Review.” JMIR Research Protocols 7, no. 1: e16. 10.2196/resprot.8836. 29348112 PMC5795095

[cre270182-bib-0035] Sai Priyanka, K. , P. Gupta , L. Gopal , et al. 2024. “Evaluation of Alveolar Bone Width Alterations Around Dental Implants.” Bioinformation 20, no. 5: 579–582. 10.6026/973206300200579. 39132240 PMC11309100

[cre270182-bib-0036] Sanz, M. , J. Lindhe , J. Alcaraz , I. Sanz‐Sanchez , and D. Cecchinato . 2017. “The Effect of Placing a Bone Replacement Graft in the Gap at Immediately Placed Implants: A Randomized Clinical Trial.” Clinical Oral Implants Research 28, no. 8: 902–910. 10.1111/clr.12896. 27273298

[cre270182-bib-0037] Schrover, R. , K. Evans , R. Giugliani , I. Noble , and K. Bhattacharya . 2017. “Minimal Clinically Important Difference for the 6‐Min Walk Test: Literature Review and Application to Morquio A Syndrome.” Orphanet Journal of Rare Diseases 12, no. 1: 78. 10.1186/s13023-017-0633-1. 28441951 PMC5405472

[cre270182-bib-0038] Seyssens, L. , C. Eeckhout , and J. Cosyn . 2022. “Immediate Implant Placement With or Without Socket Grafting: A Systematic Review and Meta‐Analysis.” Clinical Implant Dentistry and Related Research 24, no. 3: 339–351. 10.1111/cid.13079. 35313067

[cre270182-bib-0039] Seyssens, L. , A. Eghbali , and J. Cosyn . 2020. “A 10‐Year Prospective Study on Single Immediate Implants.” Journal of Clinical Periodontology 47, no. 10: 1248–1258. 10.1111/jcpe.13352. 32748983

[cre270182-bib-0040] Shahdad, S. , E. Gamble , J. Matani , L. Zhang , and A. Gambôa . 2020. “Randomized Clinical Trial Comparing PEG‐Based Synthetic to Porcine‐Derived Collagen Membrane in the Preservation of Alveolar Bone Following Tooth Extraction in Anterior Maxilla.” Clinical Oral Implants Research 31, no. 10: 1010–1024. 10.1111/clr.13648. 32799365

[cre270182-bib-0041] Sterne, J. A. C. , J. Savović , M. J. Page , et al. 2019. “RoB 2: A Revised Tool for Assessing Risk of Bias in Randomised Trials.” BMJ (Clinical Research ed.) 366: 4898. 10.1136/bmj.l4898. 31462531

[cre270182-bib-0042] Tarnow, D. P. , S. J. Chu , M. A. Salama , et al. 2014. “Flapless Postextraction Socket Implant Placement in the Esthetic Zone: Part 1. The Effect of Bone Grafting and/or Provisional Restoration on Facial‐Palatal Ridge Dimensional Change – A Retrospective Cohort Study.” International Journal of Periodontics and Restorative Dentistry 34, no. 3: 323–331. 10.11607/prd.1821. 24804283

