# Supplementary material for: Flapless Immediate Implant Placement With and Without Bone Grafting: A Systematic Review and Meta‐Analysis
Source: Clin Exp Dent Res. 2025 Jul 23;11(4):e70182. doi: 10.1002/cre2.70182 (PMC12285903; doi:10.1002/cre2.70182)
Supplement: Supplementary file 1 — Supplementary Table 1:Search Strategy (Search Date: March 30, 2024). Supplementary Tables 2. Description of excluded studies. [file CRE2-11-e70182-s001.docx]

**Appendix**

Supplementary Table 1:Search Strategy (Search Date: March 30, 2024)

| **Database** | **Search Strategy** | **Number of Record** |
| --- | --- | --- |
| Pubmed | flapless AND (“bone transplantation”[mh] OR “bone regeneration”[mh] OR “alveolar ridge augmentation”[mh] OR graft*[tiab] OR "bone transplantation*"[tiab] OR "bone regeneration*"[tiab] OR osteoconduction[tiab] OR "alveolar ridge augmentation*"[tiab] OR "alveolar ridge preservation"[tiab] OR "socket preservation"[tiab] OR "socket graft*"[tiab] OR "bone augmentation"[tiab] OR "bone replacement graft"[tiab] OR "bovine bone"[tiab] OR xenograft[tiab] OR "mandibular ridge augmentation*"[tiab] OR "maxillary ridge augmentation*"[tiab] OR "bone patellar tendon bone grafting"[tiab] OR "bone reimplantation*"[tiab] OR "bone-patellar tendon-bone grafting"[tiab] OR "ridge augmentation procedure"[tiab]) | 273 |
| Embase (embase.com) | flapless AND ('bone transplantation'/exp OR 'bone regeneration'/exp OR 'alveolar ridge augmentation'/exp OR 'graft*':ti,ab,kw OR 'bone transplantation*':ti,ab,kw OR 'bone regeneration*':ti,ab,kw OR 'osteoconduction':ti,ab,kw OR 'alveolar ridge augmentation*':ti,ab,kw OR 'alveolar ridge preservation':ti,ab,kw OR 'socket preservation':ti,ab,kw OR 'socket graft*':ti,ab,kw OR 'bone augmentation':ti,ab,kw OR 'bone replacement graft':ti,ab,kw OR 'bovine bone':ti,ab,kw OR 'xenograft':ti,ab,kw OR 'mandibular ridge augmentation*':ti,ab,kw OR 'maxillary ridge augmentation*':ti,ab,kw OR 'bone patellar tendon bone grafting':ti,ab,kw OR 'bone reimplantation*':ti,ab,kw OR 'bone-patellar tendon-bone grafting':ti,ab,kw OR 'ridge augmentation procedure':ti,ab,kw) | 288 |
| Web of Science  (Web of Science core collection 1975-Present) | TS=(flapless AND ("bone transplantation" OR "bone regeneration" OR "alveolar ridge augmentation" OR graft* OR “bone transplantation*” OR "bone regeneration*" OR osteoconduction OR "alveolar ridge augmentation*" OR "alveolar ridge preservation" OR "socket preservation" OR "socket graft*" OR "bone augmentation" OR "bone replacement graft" OR "bovine bone" OR xenograft OR "mandibular ridge augmentation*" OR "maxillary ridge augmentation*" OR "bone patellar tendon bone grafting" OR "bone reimplantation*" OR "bone-patellar tendon-bone grafting" OR "ridge augmentation procedure")) | 254 |
| Cochrane Library | flapless AND ([mh "bone transplantation"] OR [mh "bone regeneration"] OR [mh "alveolar ridge augmentation"] OR graft*:ti,ab OR (bone NEXT transplantation*):ti,ab OR (bone NEXT regeneration*):ti,ab OR osteoconduction:ti,ab OR (“alveolar ridge” NEXT augmentation*):ti,ab OR “alveolar ridge preservation”:ti,ab OR “socket preservation”:ti,ab OR (socket NEXT graft*):ti,ab OR (bone NEXT augmentation*):ti,ab OR (“bone replacement” NEXT graft*):ti,ab OR “bovine bone”:ti,ab OR xenograft:ti,ab OR (“mandibular ridge” NEXT augmentation*):ti,ab OR ("maxillary Ridge" NEXT augmentation*):ti,ab OR "bone patellar tendon bone grafting":ti,ab OR (bone NEXT reimplantation*):ti,ab OR "bone-patellar tendon-bone grafting":ti,ab OR "ridge augmentation procedure":ti,ab) | 85 |

Supplementary Tables 2. Description of excluded studies

| Reason | References |
| --- | --- |
| Study Design | MeshkatAlsadat M et al. (2022), Bungthong W et al. (2022), Amato F et al. (2018) |
| Not proper intervention or comparison | Elaskary A et al. (2022), Fernandes D et al. (2021), Naji BM et al. (2021), Kumar PRet al. (2021), Ferrantino L et al. (2021), Abd-Elrahman A et al (2020), Shahdad S et al. (2020), Grassi FRet al. (2019), Cardaropoli D et al. (2018), Natto ZS et al. (2017), Tarnow DP et al. (2014), |
| No horizontal bone changes measured | Paknejad et al et al. (2017) |
| Retracted | Bottini LP et al. (2012) |
